# Reliability and validity of the supine-to-stand test in patients with Parkinson’s disease

**DOI:** 10.1007/s11845-026-04370-7

**Published:** 2026-04-14

**Authors:** Tuba Maden, Hakan Polat, Fatih Demi̇r

**Affiliations:** 1 Department of Physiotherapy and Rehabilitation, Faculty of Health Sciences, Gaziantep University, Gaziantep, Turkey; 2 Department of Physiotherapy and Rehabilitation, Faculty of Health Sciences, SANKO University, Gaziantep, Turkey; 3 Department of Neurology, Faculty of Medicine, SANKO University, Gaziantep, Turkey

**Keywords:** Parkinson disease, Postural balance, Physical functional performance, Validation study

## Abstract

**Background:**

The Supine-to-Stand Test (SST) evaluates trunk control, muscle strength, balance, and movement coordination.

**Aims:**

This study investigated the reliability and validity of the SST in patients with Parkinson’s disease (pwPD).

**Methods:**

Forty-one pwPD participated in this cross-sectional study. Ankle plantar and dorsiflexor strength was measured with a digital dynamometer. Manual dexterity, balance, and functional mobility were assessed using the 9-Hole Peg Test (9HPT), Berg Balance Scale (BBS), and Timed Up and Go Test (TUG). To examine the convergent validity of the Supine-to-Stand Test (SST). Receiver operating characteristic (ROC) curve analysis was performed, and the optimal cut-off value was determined based on the best combination of sensitivity and specificity. Fear of falling was evaluated with the Activity-specific Balance Confidence (ABC) scale. Motor severity was assessed via the Unified Parkinson’s Disease Rating Scale Part III (UPDRS-III), and quality of life with the Parkinson’s Disease Quality of Life Scale (PDQ-39). Test-retest reliability was determined using the intraclass correlation coefficient (ICC).

**Results:**

SST test-retest reliability was excellent (ICC = 0.976, 95% CI: 0.955–0.976, *p* < 0.001). Ankle dorsiflexor strength was moderately positively correlated with SST time bilaterally (dominant *r* = 0.381, *p* = 0.014; non-dominant *r* = 0.341, *p* = 0.029). Age, disease duration, plantar strength, hand functions, rigidity, and quality of life showed no significant relation with SST (*p* > 0.05). A moderate negative correlation existed between BBS and SST (*r*=-0.341, *p* = 0.029), while TUG and SST (*r* = 0.351, *p* = 0.024) and ABC and SST (*r* = 0.384, *p* = 0.013) were moderately positively associated.

**Conclusion:**

SST is a reliable and valid tool for assessing functional performance in pwPD.

## Introduction

Parkinson’s disease is a progressive neurodegenerative disorder characterized by the loss of dopaminergic neurons, leading to a decline in motor function, postural instability, and an increased risk of falls over time [1]. Tremor, bradykinesia, rigidity, and balance disorders, frequently observed in patients, hinder motor planning and postural transitions, negatively impacting functional independence [2]. These types of motor impairments can make it difficult for a person to move from one position to another, especially to perform complex tasks such as getting up from a lying position.

Transitioning from a supine to a standing position (SST) is a functional task requiring trunk control, muscle strength, movement coordination, and dynamic balance [3]. This movement plays a key role in independence in daily living activities, being functional in situations such as getting out of bed and recovering after a fall [3]. Because SST requires an individual to assess intermuscular coordination, motor skills, and control of the center of mass, it can serve as an indicator in mobility assessments [4].

Frequent falls and the inability to get up after a fall can cause psychological and physical effects in Parkinson’s patients, making the application and applicability of functional tests such as SST extremely important [5]. In Parkinson’s patients, findings such as decreased body control and difficulty initiating movement can negatively affect SST performance, which can limit patients’ independence in daily living activities.

In this context, determining the validity and reliability of the Supine-Standing Test in Parkinson’s patients is crucial in clinical practice, both for assessing functional capacity and for planning targeted rehabilitation programs. Despite its functional importance, the SST has not been previously psychometrically evaluated in patients with Parkinson’s disease (pwPD).

The aim of this study is to investigate the usability and psychometric properties of the Supine-Standing Test in monitoring functional performance in patients with Parkinson’s disease (pwPD), and to provide practical and objective assessment in clinical practice.

## Methods

### Ethics approval

Ethical approval for the study was granted by the SANKO University Institutional Review Board (Decision no: 2024/11 − 2). All subjects were fully informed about the nature and scope of the study. Written informed consent was obtained from all subjects. The study adhered to the ethical principles outlined in the Declaration of Helsinki. This study is registered on ClinicalTrials.gov (NCT06859385).

### Participants

This cross-sectional observational study was conducted between February 2025 and June 2025 at the Research Laboratory of the Department of Physiotherapy and Rehabilitation at SANKO University. Patients were enrolled in the study through the outpatient clinic of the Department of Neurology at SANKO Hospital. A total of 41 patients with Parkinson’s disease were included in the study. Inclusion criteria were as follows: (1) diagnosis of Parkinson’s disease, (2) age over 40, (3) ability to walk with or without assistance, (4) no change in treatment regimen in the past two months, (5) voluntary participation in the study, and (6) no verbal or cognitive communication barriers. Exclusion criteria included the presence of orthopaedic, psychiatric, or other neurological conditions.

### Data collection tools

Initial assessments included demographic and clinical characteristics, SST, muscle strength measurements (ankle dorsiflexors and plantar flexors), Nine-Hole Peg Test (9HPT) for dominant and non-dominant sides, Berg Balance Test (BBS), Timed Up and Go (TUG), Activity-Specific Balance Confidence (ABC) Scale, and Parkinson’s Disease Quality of Life Scale (PDQ-39). The severity of motor symptoms was assessed using the Unified Parkinson’s Disease Rating Scale Part III (UPDRS-III).

All assessments were performed while patients were in their ON period, and stability was confirmed through careful monitoring of symptoms and physical examination.

A second SST was administered one week later by the same therapist, and the SST completion time was used for reliability analysis. To detect stability and short-term changes in the measured variables, a one-week interval was given between the two consecutive tests. Test-retest reliability studies typically use a one-week interval to capture any changes in performance while minimizing memory effects or fluctuations in participants’ condition. During the interim period, each patient’s on/off-period symptoms and physical condition were carefully monitored to ensure stability. A physical examination was conducted to confirm stability and compared with the participant’s initial assessments.

### Assessments

#### SST

The SST was used to assess the participants’ ability to rise from a supine position. The patient was placed in a supine position, without shoes, on a soft mat in a quiet laboratory environment. The patient was asked to stand up upon the command “start”. The time taken to complete the task was recorded in seconds. The average duration from two repeated trials used for analysis [6, 7]. Longer SST completion times indicate greater impairments in functional mobility.

 These assessments were conducted to examine the convergent validity of the Supine-to-Stand Test (SST).

#### Muscle strength measurements

Muscle strength of the ankle dorsiflexors and plantar flexors was assessed using a digital hand dynamometer (Commander Muscle Tester, JTech, Midvale, USA). Ankle muscle strength has been identified as a potential predictor of walking capacity in neurological disorders [8, 9]. While the dynamometer was positioned anteriorly and posteriorly to the head of the metatarsal bones, respectively, pwPD performed a 3-second maximal isometric voluntary contraction for both the ankle dorsiflexors and plantar flexors [10]. The strength of ankle flexor muscles was measured separately on both sides, with each test performed twice in the supine and prone positions, respectively. The average of the two trials was recorded in Newtons (N).

#### 9HPT

Fine manual dexterity of the participants was assessed using the 9HPT. The 9-Hole Peg Test (9HPT) was selected to assess fine manual dexterity as it is a well-validated, reliable test that reflects upper limb function, which is functionally relevant to the Supine-to-Stand Test (SST) performance. To complete the task, the patient was instructed to insert the pegs into the holes of the board while seated. The test was performed bilaterally, with two trials for each hand, with the goal of completing the task as quickly as possible. The average time taken to complete the test was recorded. As the disease progresses, patients typically experience a decline in manual dexterity. The 9HPT demonstrates strong convergent validity with other tests of manual dexterity and upper limb function, showing high reliability both within and between test sessions. It also effectively distinguishes between healthy people and patients with different levels of upper limb disability [11, 12].

#### BBS

The BBS is a functional measure of balance widely employed in pwMS. It consists of 14 functional activities commonly used in daily life (sitting unsupported, standing unsupported, sitting to standing, standing to sitting, standing with feet together, standing with eyes closed, transfers, reaching forward while standing, picking up an object from the floor turning 360 degrees, turning to look behind, standing with one foot in front, placing alternate foot on stool, and standing on one leg). Each task is scored on a scale of 0 to 4; 0 indicates failure to complete the task, and 4 indicates independent performance. Total possible scores range from 0 to 56. Lower scores indicate greater balance impairment. Scores between 0 and 20 points indicate severe balance impairment, 21–40 points indicate acceptable balance, and 41–56 points indicate good balance [12, 13].

#### TUG

The TUG test is used to assess functional mobility in pwPD [14]. During the test, the patient was asked to get up from the chair, walk three meters at a pace of their choosing and at a comfortable speed, then return and sit back down. The test duration was recorded in seconds. As the duration increased, the patient’s performance deteriorated.

#### ABC

The ABC scale is a self-report measure of balance confidence, used to assess fear of falling during 16 activities of daily life (both outdoors and indoors). Items are rated on a scale that ranges from 0 (no confidence) to 100 (full confidence). Higher scores indicate greater confidence in performing activities [15].

#### UPDRS-III

The Unified Parkinson’s Disease Rating Scale Part III (UPDRS-III) was used to assess the motor severity of Parkinson’s disease. The UPDRS-III is a sub-parameter of the Parkinson’s Disease Rating Scale (UPDRS) used to assess motor symptoms such as rigidity, postural stability, tremor, bradykinesia, and gait. Each item is scored on a scale from 0 = normal to 4 = severe. A total score indicates general motor impairment, while higher scores indicate more severe functional impairment [16].

#### PDQ-39

A Parkinson’s disease-specific quality of life questionnaire was used to assess quality of life. The questionnaire is a general assessment tool that includes eight sub-dimensions, including cognitive, emotional, physical, and social domains. Higher scores indicate lower quality of life [17].

### Statistical analysis

Statistical analyses were performed using SPSS version 25.0 (IBM Corp., Armonk, NY). Descriptive statistics of the parameters are expressed as mean ± standard deviation (X̄ ± SD). The normality of the data distribution was examined using the Kolmogorov-Smirnov test.

A two-way random effect and intraclass correlation coefficient (ICC) absolute fit model was used to assess test-retest reliability. A 95% confidence interval (95% CI) was applied for measurement variability. Excellent reliability is represented by an ICC value of 1.0, while lack of reliability is represented by a value of 0.0. An ICC value of ≥ 0.90 indicates excellent reliability [18].

The following formulae were used to determine the minimal detectable change (MDC) and the standard error of measurement (SEM) [19]:

$$\:SEM=SD\:\sqrt{1-ICC}\:\:\:\:\:\:\:\:\:\:\:\:\mathrm{a}\mathrm{n}\mathrm{d}\:\:\:\:\:\:\:\:\:MDC=SEM\mathrm{x}1.96\mathrm{x}\sqrt{2}$$

where SD represents the pooled standard deviation.

The SEM measured within-participant variability by reflecting the measurement error of the instrument [20]. The MDC, a more moderate estimate of accuracy, is the smallest observable variation beyond measurement error [21].

Convergent validity was assessed through correlation analysis between the SST and age, disease duration, muscle strength, 9HPT, BBS, TUG, ABC Scale, UPDRS-III and PDQ-39. Pearson’s or Spearman’s correlation coefficient was used to analyze relationships between variables, as appropriate. Correlation coefficients were interpreted as strong (> 0.50), moderate (0.30 to 0.50), or weak (0.20 to 0.30) [22]. A p-value of < 0.05 was considered statistically significant.

By examining the deviation of the mean difference and bias, a Bland-Altman plot was created to evaluate the agreement between the SST test-retest results. Additionally, simple linear regression analysis was used for bias verification.

The clinical cut-off value of the SST was established using the receiver operating characteristic (ROC) curve analysis. The highest acceptable sensitivity and specificity were combined to determine the optimal cutoff (significant limit) value.

G*Power version 3.1.9 (Heinrich Heine University, Germany) was used for power analysis. A significant association between SST and TUG has been reported in stroke patients (*r* = 0.461, *p* = 0.001) (Ng et al., 2023). Our current study requires at least 34 participants (alpha = 0.05, power = 0.80). Another study found a significant association between SST and TUG (*r* = 0.461, *p* = 0.001) (Ng et al., 2023).

## Results

Demographic and clinical properties of pwPD with a mean age of 67.41 ± 10.84 and a disease duration of 8.70 ± 5.61 are given in Table 1.

**Table 1:** Demographic and clinic properties in pwPD

Variables	Mean ± SD
Age (Years)	67.41 ± 10.84
Gender, n (%)	
Female/Male	22/19
Disease Duration, years	8.70 ± 5.61
Hoehn and Yahr Scale, score (1–5), n (%)	
1	3 (% 7.3)
2	26 (% 63.4)
3	12 (% 29.3)
4	-
Rigidity (n)	
0	12
1	17
2	12
Muscle Strength, Newton	
Dominant Ankle Dorsiflexor	31.00 ± 7.53
Nondominant Ankle Dorsiflexor	32.42 ± 7.29
Dominant Plantar Flexor	36.27 ± 8.87
Nondominant Plantar Flexor	38.00 ± 8.21
Nine Hole Test, seconds	
Dominant	32.63 ± 12.44
Nondominant	35.97 ± 13.45
Berg Balance Test, score	34.19 ± 9.37
Timed Up and Go test, sn	23.39 ± 6.90
ABC Scale, score	58.95 ± 9.15
PDQ-39	34.57 ± 7.40

*SD* standard deviation, *RRMS* Relapsing-Remitting MS, *SPMS* Seconder Progressive MS, *ABC Scale*: Activity-specific Balance Confidence Scale, *PDQ-39*: Parkinson’s Disease Quality of Life Scale

The test-retest reliability of the SST in pwPD was excellent, as evidenced by an ICC of 0.976 (95% CI: 0.955–0.976, *p* < 0.001). The SEM was 0.90 and MDC was 1.34 s (Table 2). These MDC% and SEM% values are well within the recognized clinical measurement reliability standards [23]. An improvement of more than 1.34 s indicates meaningful clinical change.

**Table 2:** Test-retest reliability of the SST in pwPD

Variable	Trial 1 Mean ± SD	Trial 2 (re-test) Mean ± SD	ICC	95% CI	*p*	SEM	MDC
SST	18.14 ± 5.69	17.68 ± 5.93	0.976	0.955–0.976	< 0.000*	0.90	1.34

*p < 0.05, *SST* Supine-to-Stand Test, *ICC* Intraclass Correlation Coefficient, *SD* Standard Deviation, *CI* Confidence Interval, *SEM* Standard Error of Measurement, *MDC* Minimal Detectable Change

Ankle dorsiflexor strength was positive moderately correlated with SST time, bilateraly (*p* < 0.05) (Table 3). Age, disease duration, plantar strength, hand functions, rigidity and quality of life had no significant relationship with SST (*p* > 0.05) (Table 3). A negative moderate connection was established between the Berg Balance Test and SST (*r*=−0.341, *p* = 0.029). There was a moderately positive association between the Timed Up and Go test and SST (*r* = 0.351, *p* = 0.024). A positive, moderately significant association was discovered between the Activity-specific Balance Confidence Scale and SST (*r* = 0.384, *p* = 0.013) (Table 3).

**Table 3:** Correlations between SST and demographic, clinical, and functional measures in pwPD

Variable		Age	Disease Duration	Ankle Dorsiflexor		Plantar Flexor		9HPT		BBS	TUG	ABC	UPDRS-III	PDQ-39
				D	ND	D	ND	D	ND					
SST	r	0.164	0.214	0.381	0.341	−0.207	−0.004	0.110	0.153	−0341	0.351	0.384	0.910	0.006
	p	0.307	0.178	0.014*^a^	0.029*^a^	0.194^a^	0.981^a^	0.494^a^	0.339^a^	0.029*^a^	0.024*^a^	0.013*^a^	< 0.000^b^	0.972^a^

*p < 0.05; ^a^ Pearson correlation analysis was performed to the relationship of parameters, ^b^ Spearman correlation analysis was performed to the relationship of parameters. 9HPT: The Nine Hole Peg Test, BBS: Berg Balance Test, TUG: Timed Up and Go test, ABC: Activity-specific Balance Confidence Scale; UPDRS-III: The Unified Parkinson’s Disease Rating Scale Part III, PDQ-39: Parkinson’s Disease Quality of Life Scale D: Dominant hand; ND: Non-dominant hand

**Fig. 1:**
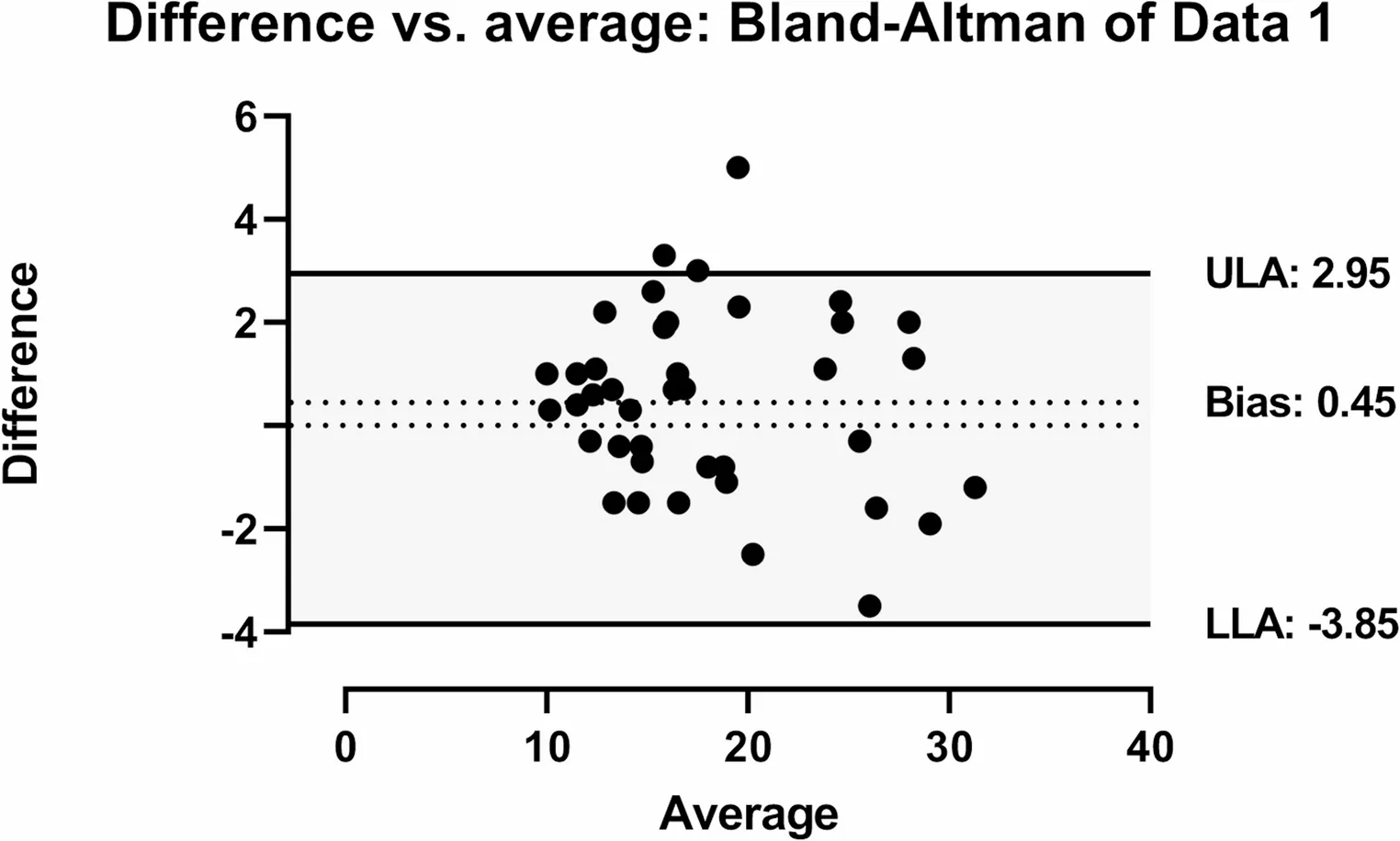
Bland-Altman plot showing the upper and lower limits of agreement (ULA and LLA, 95%) between test-retest scores of the SST

The upper and lower limits of the SST test are shown in the Bland-Altman plot in Fig. 1. The upper and lower limits of the test in Parkinson’s patients were determined to be 2.95 and − 3.85, respectively. According to the results of the linear regression analysis, a bias of 0.45 was detected (β = 0.042, *p* = 0.389).

**Fig. 2:**
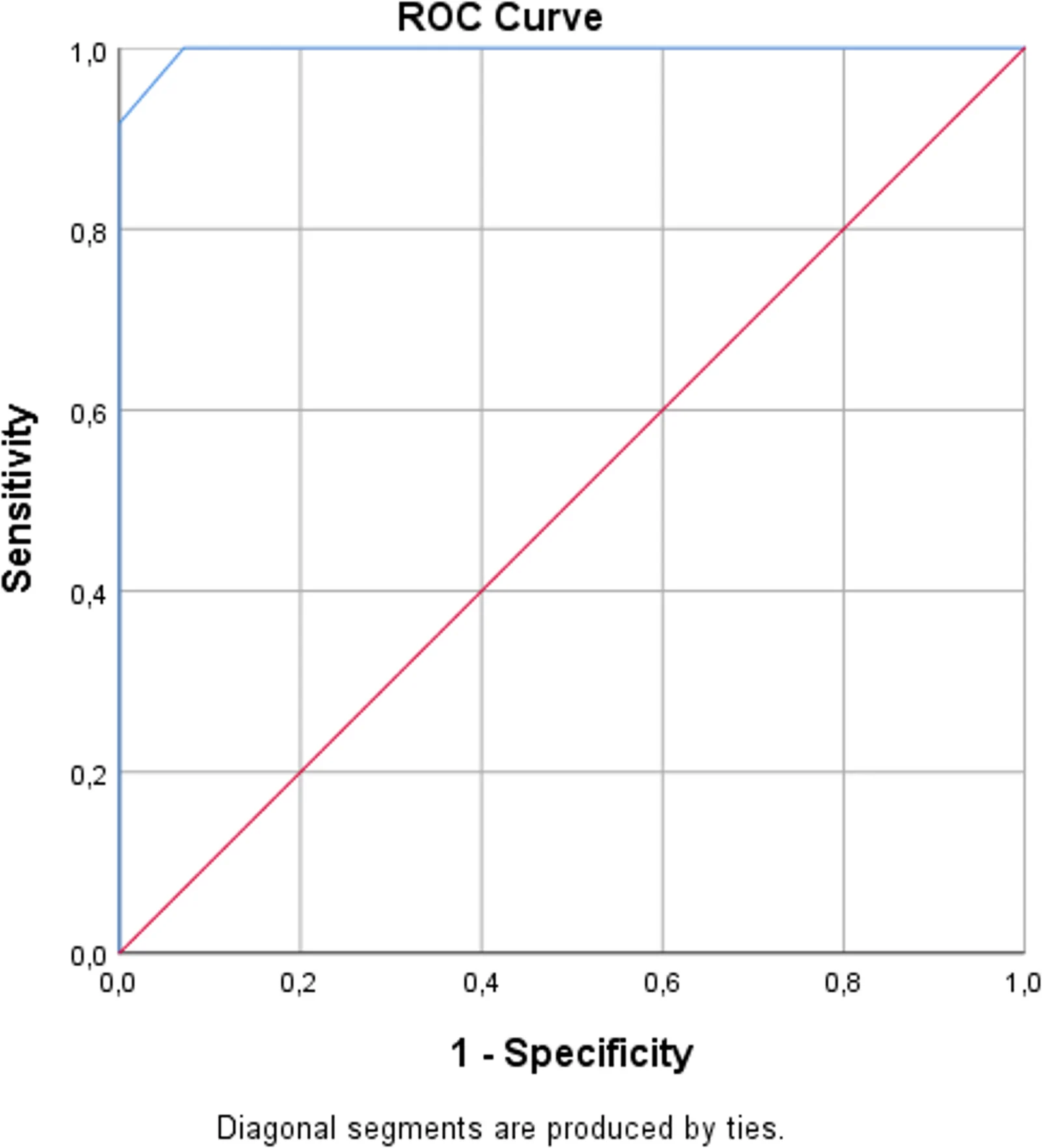
Roc-curve for SST

A clinical cut-off score of 18.70 for SST in pwPD was established using ROC analysis. For SST, scores of 18.70 or below, with 95% confidence, were regarded as normal (95% confidence interval lower bound: 0.985, upper bound: 1.000; area under the curve: 0.997; *p* < 0.000) (Fig. 2). Table 4 displayed the SST’s sensitivity and specificity.

**Table 4:** The sensitivity, specifity and cut-off point of SST

	AUC	95% CI	Cut-off point	p	Sensitivity (%)	Specificity (%)
SST	0.997	0.985–1.000.985.000	18.70	0.000	100.0	92.9

*SST* Supine-to-stand Test, *AUC* Area under the curve, *CI* Confidence interval

## Discussions

This study demonstrated that the SST is a valid and reliable measure of functional motor competence in pwPD.

Bradykinesia, rigidity, postural instability, and decreased muscle strength, which are motor symptoms of Parkinson’s disease, cause functional problems in daily living activities. In SST, it is a functional status that involves the action of rising from a supine position to a standing position. This is one of the activities that individuals perform every day. Furthermore, the SST functional status is critically important in terms of recovery after falls, a common symptom in Parkinson’s patients. Therefore, the SST provides valuable information regarding the capacity to perform movement transitions in Parkinson’s patients.

In our study examining the validity and reliability of the SST test in individuals with Parkinson’s disease, our test-retest reliability was excellent (ICC = 0.976). This result indicates that the SST test provides consistent and similar results across applications. The low SEM (0.90 s) and MDC (1.34 s) values indicate that the measurement error of the test is minimal and demonstrate its measurement accuracy. Reliability values at this level are very important in clinical practice, as healthcare professionals need to be able to accurately assess, apply and monitor motor performance. Furthermore, the ease of implementation, short completion time, and applicability in any setting of the SST emphasise that it is a practical assessment tool for evaluating the motor performance of Parkinson’s patients.

When applied to people with Parkinson’s disease (pwPD), the 1.34-second MDC value indicates that changes above this threshold in the SST can be interpreted as genuine improvements beyond measurement error [23, 24]. The low SEM value (0.90 s) further confirms the test’s sensitivity and specificity in detecting meaningful changes over time [23].

Although it has been demonstrated that SST performance increases with age in other populations, such as healthy adults [25], no significant relationship between age and SST was found in our Parkinson’s group; this suggests that age may have a less pronounced effect on standing up from a supine position in pwPD, primarily due to the motor symptoms of the disease.

Previous studies have reported excellent reliability for SST, with ICC values ranging from 0.946 to 1.000 across different populations, including individuals after stroke, the most common neurological disorder [3]. Although slightly higher reliability values have been reported, our findings demonstrate that SST yields consistent and clinically meaningful results in pwPD, despite the higher motor symptom involvement and variability specific to this population.

The validity of the SST in Parkinson’s disease patients (pwPD) has been determined in relation to the fundamental parameters required for this activity and its applicability in Parkinson’s disease patients. These parameters are currently primarily balance, fall status, and ankle muscle strength values. These parameters are supported by correlations with established clinical measurements frequently used in Parkinson’s disease, such as the Berg Balance Scale (BBS), the Timed Up and Go (TUG) test, the Activity-Specific Balance Confidence (ABC) scale, and ankle dorsiflexion muscle strength.

Moderate correlations were observed between SST duration and these measurements, confirming the convergent validity of SST as an indicator of functional motor competence in pwPD [3]. Specifically, a negative moderate correlation was observed with BBS (*r* = −0.341) and positive moderate correlations with TUG (*r* = 0.351) and the ABC scale (*r* = 0.384) indicate that longer SST durations are associated with poorer balance, reduced mobility, and low confidence in performing daily activities. No significant relationship was found between SST duration and age, disease duration, plantar muscle strength, hand function, rigidity, or quality of life; this highlights the predominant profile of pwPD’s unique motor symptoms. These findings reinforce the clinical utility of SST in assessing functional performance and fall risk in Parkinson’s disease patients.

Balance problems are common in pwPD and have been associated with an increased risk of falls due to postural instability and impaired motor control. Multiple studies have demonstrated that clinical balance and mobility measures such as the Berg Balance Scale (BBS), Timed Up and Go (TUG), and Activities-specific Balance Confidence (ABC) scale are important indicators of fall risk in Parkinson’s disease, highlighting the relevance of evaluating functional postural transitions in this population (BBS, TUG, and ABC scale have established psychometric properties in PD [24]. Successful transitioning from supine to standing requires coordinated balance mechanisms, and although the SST has not yet been widely validated as a direct predictor of falls, our observed moderate correlations between SST and these balance-related measures suggest that slower SST times are associated with poorer balance and mobility performance in pwPD. Moreover, self-reported balance confidence and clinical measures have been shown to predict recurrent falls, reinforcing the clinical significance of assessing both performance-based and self-reported functional balance in Parkinson’s disease [25]. These findings support the conceptual interpretation of the SST as reflecting functional motor competence relevant to fall risk and postural control, and highlight the need for future prospective studies to determine whether supine-to-stand performance can reliably predict actual falls in pwPD.

The TUG and measures of gait speed assess mobility in activities of daily living and reflect functional capacity in people with Parkinson’s disease. While the TUG evaluates short-distance mobility and functional balance, gait speed provides information about walking capacity and overall mobility performance in pwPD, with gait speed recognized as a meaningful indicator of functional ability and independence.

Tests evaluating gait parameters in Parkinson’s disease (PD) patients have demonstrated both reliability and validity with functional performance measurements, supporting their clinical significance [26]. Although SST has not been comprehensively addressed as a direct predictor of walking speed in PD, the moderate correlation between SST duration and TUG performance in our study (*r* = 0.351) suggests that longer SST durations may reflect mobility impairments associated with walking and balance domains.

This relationship parallels findings in other neurological populations where correlations have been identified between SST duration and functional mobility measures; for example, the relationship between TUG performance and SST duration in post-stroke individuals supports the potential of SST as an indirect indicator of broader motor competence in pwPD [3].

These results reinforce the clinical benefit of integrating multiple functional assessments, such as SST, gait and balance scales, to capture the multifaceted nature of mobility impairment in Parkinson’s disease.

In this study, the SST completion time showed a moderate correlation with the dorsiflexor muscle strength of both lower extremities. This reflects the importance of lower limb muscles in standing up from a supine position in pwPD. However, the limited correlations with muscle strength suggest that SST performance is influenced not only by isolated muscle strength but also by integrated requirements such as trunk control, dynamic balance, and postural coordination [23].

The asymmetric presentation of motor symptoms in Parkinson’s disease may explain differences in SST performance between the more affected and less affected sides and highlights the importance of considering lateralised motor impairment when interpreting results [27].

Unlike fine motor tasks, which rely on distal upper extremity function, SST primarily assesses gross motor skills, balance, and functional mobility; it emphasises whole-body integration rather than isolated extremity strength.

The absence of a strong correlation between SST performance and PDQ-39 scores assessing quality of life in our study suggests that quality of life in Parkinson’s disease stems not only from physical performance measurements but also from its multidimensional nature [28, 29].

In future studies, it is recommended that the relationship between SST performance and other functional domains, risk of falling, and quality of life outcomes in Parkinson’s disease patients be investigated to further validate its clinical validity.

Our sample size limited correlation analyses due to the concentration of motor symptoms within a specific range. At this point, in order for the results to be generalised, it is necessary to test the study on samples with different disease severities and a wide range of motor impairments. Furthermore, the failure to examine and investigate symptoms such as difficulty initiating movement or freezing phenomena as confounding factors is a significant limitation of the study.

## Conclusions

The findings from this study underscore the potential of the SST as a simple, time-efficient, and clinically relevant tool for evaluating functional motor competence in pwPD. Its moderate correlations with established mobility and balance assessments, including the TUG and BBS, suggest that clinicians may consider integrating the SST into routine evaluations to screen for functional impairment, monitor disease progression, and identify individuals at risk of falls. Furthermore, the SST could be incorporated into rehabilitation programs as a target outcome to track improvements in functional mobility over time. Future prospective studies are warranted to confirm its utility in predicting future disability, fall risk, and the effectiveness of specific therapeutic interventions in Parkinson’s disease.

## Declarations

### Clinical Trial No

NCT06859385.
